# Recent Advances in Knowledge Representation and Management in Healthcare: the Emerging Role of Large Language Models

**DOI:** 10.1055/s-0046-1816630

**Published:** 2026-11-03

**Authors:** Catalina Martínez Costa, Jean Charlet, Licong Cui

**Affiliations:** 1 Department of Informatics and Systems, University of Murcia, CEIR Campus Mare Nostrum, IMIB-Arrixaca, MurciaSpain; 2 Sorbonne Université, INSERM, Univ Sorbonne Paris Nord, LIMICS, ParisFrance; 3 AP-HP, DRCI, ParisFrance; 4 McWilliams School of Biomedical Informatics, The University of Texas Health Science Center at Houston, Houston, TXUSA

**Keywords:** Knowledge representation and management, ontology, knowledge graph, large language model, International Medical Informatics Association

## Abstract

**Objectives** : To select and summarize the best papers published in 2024 in the field of Knowledge Representation and Management (KRM) for inclusion in the International Medical Informatics Association (IMIA) Yearbook.

**Methods** : We conducted PubMed queries and followed the IMIA Yearbook guidelines for performing biomedical informatics literature review to select the best papers in KRM published in 2024.

**Results** : We retrieved 2,071 publications from PubMed, from which 14 were selected as candidate best papers. After in-depth evaluation, three were selected as the best papers for the KRM section. The candidate papers covered four main thematic areas: (i) Ontology Development and Application, (ii) Knowledge Graph Construction and Completion, (iii) Large Language Model (LLM)-Augmented Knowledge Representation and Reasoning, and (iv) Semantic Interoperability and Data Harmonization. Two of the selected best papers address advances in knowledge graph construction and completion, while the third focuses on semantic interoperability and harmonization across health data standards.

**Conclusions** : The selection process for the best papers in the KRM section for 2024 showed a wide spectrum of topics. Across several papers, it can be observed the emerging role of LLMs in enhancing ontology engineering, medical knowledge extraction, and reasoning, especially when integrated with structured clinical data and formal knowledge representations.

## 1. Introduction

Knowledge Representation (KR) focuses on the formal modelling of information about the world in a way that a computer system can use it to perform complex tasks such as reasoning and decision-making. In the biomedical domain, the growing volume and diversity of available data, including electronic healthcare records (EHRs), genetic data, medical images and sensor outputs, requires multimodal data integration [ 1 ]. This presents significant representational challenges, as systems must be able to capture, integrate and reason across heterogeneous data types. Effective KR frameworks are essential for enabling the efficient reuse of biomedical knowledge, supporting interoperability, and driving advancements in areas such as personalized medicine [ 2 ], clinical decision support [ 3 ], and biomedical research [ 4 ].

Knowledge management (KM) refers to the processes and tools used to acquire, store, share and maintain knowledge effectively. It ensures that accurate and relevant information is accessible to the appropriate systems or individuals at the right time, thereby enhancing decision-making, collaboration, and organizational learning. Recent progress in biomedical KM includes the automatic construction and updating of knowledge graphs [ 5 6 ], the development of semantic technologies to support the discovery, integration, and reuse of biomedical knowledge across systems [ 7 , 8 ], and the application of advanced techniques such as link prediction to infer new relationships, and temporal knowledge modeling to capture the evolution of knowledge over time [ 9 , 10 ].

## 2. Paper Selection Method

To identify the most significant contributions in the Knowledge Representation and Management (KRM) field published in 2024, we searched PubMed using the same query set and Medical Subject Headings (MeSH) descriptors applied in recent years [ 11 ]. Our inclusion criteria focused on original research articles published between January 1, 2024, and December 31, 2024, excluding publication types such as reviews, editorials, comments, case reports, and letters to editors. This search yielded 2,071 publications.

We followed the usual three-stage process [ 12 ] typically employed by the IMIA Yearbook sections for selecting the best papers. First, KRM section editors conducted an initial screening of articles based on titles, abstracts and publication types. The Rayyan web-based tool for systematic reviews [ 13 ] was used to facilitate the selection process. From this initial screening, 178 papers were identified for further evaluation.

In the second stage, a more detailed review was performed by the KRM section editors to select a subset of candidate best papers for peer evaluation. This stage focused on the innovativeness, scientific and/or practical impact, and methodological quality of the papers. As a result, 14 papers were selected as candidate best papers [ 5 6 7 8 9 10 , 14 15 16 17 18 19 20 21 ].

Each candidate paper was subsequently assessed by at least four reviewers, including KRM section editors, IMIA chief editors, and external experts in KRM. The evaluation criteria included the significance of the topic, scientific and practical impact, scientific content, originality, literature quality, and presentation quality. The final selection of best papers was determined during a meeting of the entire editorial board, informed by the reviewers' evaluations and the section editors' reports. As a result, three papers were chosen as the best.

## 3. Results

### 3.1 Best Papers for 2024

From the 14 candidate best papers, the three listed in Table 1 were selected as best papers. Further content summaries of the three best papers are provided in the appendix of this synopsis.

**Table 1.** Best paper selection of articles for the IMIA Yearbook of Medical Informatics 2025 in the section ‘Knowledge Representation and Management’. The articles are listed in alphabetical order of the first author's surname

Section Knowledge Representation and Management
• Postiglione M, Bean D, Kraljevic Z, Dobson RJ, Moscato V. Predicting future disorders via temporal knowledge graphs and medical ontologies. IEEE J Biomed Health Inform 2024;28(7):4238-4248.
• Xiao Y, Zhang S, Zhou H, Li M, Yang H, Zhang R. FuseLinker: Leveraging LLM's pre-trained text embeddings and domain knowledge to enhance GNN-based link prediction on biomedical knowledge graphs. J Biomed Inform 2024;158:104730.
• Zhang S, Cornet R, Benis N. Cross-Standard Health Data Harmonization using Semantics of Data Elements. Sci Data 2024;11(1):1407.

The first article, authored by Postiglione *et al.* [ 10 ], presents MedTKG, a temporal knowledge graph (KG) framework designed to predict future medical disorders by integrating temporal KGs with medical ontologies. A key innovation of this work is the incorporation of temporal dynamics into KGs, a relatively underexplored area. The authors model a patient's medical history as a sequence of time-stamped KGs, while a static graph is used to model the hierarchies of concepts extracted from domain ontologies. To enable prediction of future disorders, the framework uses a relation-aware Graph Convolutional Network (GCN) that captures different kinds of medical relationships between diseases, symptoms, and treatments. Temporal evolution is modelled using gated recurrent units, and a static graph constraint is introduced to maintain alignment with ontology-based semantic structures. The method was evaluated using the MIMIC III dataset and compared against ten state-of-the-art disorder prediction models. MedTKG demonstrates improved performance compared to baseline models, highlighting the value of combining structured temporal data with semantic enrichment from ontologies. The work has potential applications in early diagnosis, preventive healthcare, and clinical decision support, and shows the potential of integrating ontologies in temporal KGs for predicting future disorders.

In the second paper, Xiao *et al.* [ 9 ] developed FuseLinker, a novel link prediction framework for biomedical KGs (BKGs). FuseLinker integrates graph structural information, domain knowledge, and pre-trained textual embeddings from large language models (LLMs) to enhance the performance of graph neural network (GNN) based link prediction. The framework was evaluated against traditional KG embedding models and GNN-based approaches across four public BKG datasets, consistently outperforming all baselines. In addition, the framework was also validated in a drug repurposing case study to predict diseases that can be treated with a particular drug, and drugs that can potentially treat Parkinson's disease. FuseLinker is a novel link prediction framework that exploits both KG structure and domain knowledge information and shows significant potential for practical applications in biomedical and clinical tasks.

The third article, authored by Zhang *et al.* [ 8 ], developed a novel approach for harmonizing data elements across diverse health data standards. The authors adopted the SSSOM (Simple Standard for Sharing Ontological Mappings) framework to define and reuse semantic mappings, which are represented in the Resource Description Framework (RDF) language. This representation facilitates the structured capture of key information necessary for harmonization across standards. To evaluate their approach, the authors implemented a small-scale demonstration involving 64 data elements from five health data standards and nine topic areas, with queries tested at varying levels of granularity. Although the scope of validation is limited and the mappings have yet to be reviewed by a broader community, the study demonstrates the feasibility of a concept-based, semantic harmonization methodology. It provides a promising foundation for advancing interoperability in health information systems.

The candidate best papers can be grouped into four major categories: (1) Ontology Development and Application; (2) KG Construction and Completion; (3) LLM-augmented Knowledge Representation and Reasoning; and (4) Semantic Interoperability and Data Harmonization.

### 3.2 Ontology Development and Application

Three articles [ 14 15 16 ] fall under the ontology development and application category, each contributing to data integration and clinical decision support in distinct medical domains.

In [ 14 ], Yu *et al.* developed PCAO2, an ontology designed to integrate genotypic, phenotypic, and lifestyle data for prostate cancer (PCa). Built using Web Ontology Language (OWL) and validated by experts, it incorporates knowledge from existing ontologies, clinical resources, and literature. PCAO2 supports tools like PCaTreP for precision medicine and PCLiON for lifestyle guidance, helping clinicians classify patients and plan treatments. Freely available online, it addresses the lack of specialized cancer ontologies and serves as a model for future developments.

Xia *et al.* [ 15 ] developed WWECA, an ontology designed to support the management of epilepsy in women of reproductive age by addressing key issues like medication effects on fertility, preconception counseling, and pregnancy-related risks. Built using the Basic Formal Ontology (BFO), it classifies and organizes terms, sourced from MeSH, National Cancer Institute (NCI) Metathesaurus, literature, and clinical guidelines, into a structured hierarchy. Each entity is annotated with attributes such as definitions, synonyms, and Chinese labels, and formalized in OWL. An associated online Question-answering system supports clinical decision-making, and expert evaluations affirm its value. WWECA is publicly available on the NCBO BioPortal.

Faggioli *et al.* [ 16 ] introduced the BrainTeaser Ontology (BTO), developed to model and integrate clinical data for rare neurological diseases, specifically Amyotropic Lateral Sclerosis (ALS) and Multiple Sclerosis (MS), to support disease progression prediction and enhance data interoperability. Built on standard ontologies such as NCI Thesaurus, SNOMED CT, and Unified Medical Language System (UMLS), BTO supports disease progression prediction, explainable artificial intelligence, and semantic querying through SPARQL. Its modular design allows for future expansion to conditions like Parkinson's and Alzheimer's, and integration of multimodal data sources.

### 3.3 Knowledge Graph Construction and Completion

This category includes two of the best papers [ 9 , 10 ], highlighting significant advances in the field of KG construction and completion.

In [ 10 ], MedTKG introduces a temporal knowledge graph framework enriched with ontologies to predict future medical disorders, while in [ 9 ], FuseLinker enhances GNN-based link prediction by fusing domain knowledge with LLM-derived text embeddings. The first work addresses the representation of temporal data in KGs, a relatively underexplored area, while FuseLinker contributes to KG completion. Both demonstrate the power of combining semantic information with structural and temporal graph dynamics for improved clinical inference and prediction.

Also focused on KG completion, Wang *et al.* [ 5 ] propose KDGene, a framework that constructs a biological KG centered on diseases and genes, addressing the limitations of existing methods that often overlook complex inter-entity relationships. KDGene incorporates an interaction module within tensor decomposition to bridge entity and relation embeddings, improving the representation of semantically similar concepts. This enables more accurate disease-gene prediction and outperforms traditional KG embedding models on benchmark datasets.

In [ 17 ], Sousa *et al.* address the lack of explainability when using standard embedding methods for representing data as KGs to be used by machine learning models. The authors present KGsim2vec, a method for generating explainable vector representations by capturing different semantic aspects from the KG. They apply it to predict protein-protein interactions, and it improves both predictive performance and transparency, outperforming black-box methods based on standard KG embeddings or GNNs.

In [ 18 ], Devarakonda *et al.* constructed a KG from clinical trial data and applied inductive inference using KG embeddings to predict missing links, particularly for unseen nodes. This enabled recommendations for clinical trial elements like cohort definitions and objectives. It achieved relevance scores between 70% and 83%, as measured by text similarity to actual clinical trial components. This illustrates the potential of inductive KG embedding methods to effectively complete and extend KGs in the biomedical domain.

### 3.4 LLM-Augmented Knowledge Representation and Reasoning

Three articles [ 6 , 7 , 19 ] fall into this category, showing how LLMs can enhance ontology engineering, medical knowledge extraction and diagnostic reasoning through the integration with structured knowledge sources.

In [ 6 ], the authors present AutoRD, an LLM-based system designed for rare disease information mining. AutoRD extracts structured information about rare diseases and constructs corresponding KGs from unstructured medical texts. The system uses GPT-4, enhanced with domain-specific knowledge from rare disease and phenotype ontologies such as Orphanet and the Human Phenotype Ontology. By integrating ontology-driven prompting and reasoning, AutoRD improves the accuracy of entity and relation extraction. The system was evaluated on a rare disease benchmark dataset, demonstrating notable improvements in both entity recognition and relationship extraction tasks, highlighting its potential to support rare disease research and clinical decision-making.

In [ 7 ], Toro *et al.* propose DRAGON-AI, a system that uses LLMs for automated ontology generation. DRAGON-AI dynamically generates both textual definitions and logical relationships by drawing on existing ontologies and unstructured text sources using a retrieval-augmented generation (RAG) framework. While it does not replace expert curation, DRAGON-AI effectively supports ontology editors by reducing manual effort and accelerating the ontology development process, particularly in generating consistent and contextually relevant content.

In [ 19 ], Afshar *et al.* explore the impact of integrating UMLS into LLMs for diagnosis generation. They find that while LLMs have some inherent medical knowledge, their diagnostic accuracy improves significantly with UMLS support. However, evaluation revealed a weak correlation between automated metrics and expert assessments, highlighting the need for better-aligned evaluation methods.

### 3.5 Semantic Interoperability and Data Harmonization

In addition to the best paper [ 8 ], two candidate papers [ 20 , 21 ], address key challenges in achieving semantic interoperability and harmonizing heterogeneous health data across systems, standards, and domains.

Tejani *et al.* [ 20 ] propose a framework that standardizes radiology findings by integrating common data elements with HL7 FHIR structures and aligning them with established ontologies such as RadLex, SNOMED CT, and LOINC. The approach is demonstrated through the encoding of pulmonary nodules on chest CT scans, showing its ability to harmonize imaging data. This method promotes semantic consistency, facilitates data exchange, and supports advanced clinical decision-making across healthcare systems.

Ohlsen et al. [ 21 ] introduce PCEtoFHIR, a framework for integrating post-coordinated SNOMED CT expressions (PCEs) into HL7 FHIR resources. PCEs allow for rich clinical detail by combining multiple SNOMED CT concepts, but their complexity complicates storage and interoperability in FHIR-based systems. The authors address this by using the OWL representation of SNOMED CT to decompose PCEs into constituent components, which are then mapped to appropriate FHIR resource elements. This approach offers a viable solution for representing complex clinical concepts in a semantically interoperable format, supporting advanced clinical data exchange and analysis.

## 4. Conclusions

The 14 candidate best papers selected for the KRM section in the year 2024 cover diverse topics. Three papers [ 14 15 16 ] focus on ontology development and application, each contributing to data integration and clinical decision support within specialized medical domains through the creation of domain-specific ontologies. In the area of KG construction and completion, two best papers [ 9 10 ] and additional contributions [ 5 , 17 , 18 ] demonstrate significant progress in using ontologies, embeddings, and LLMs to enhance link prediction, KG construction and explainability within biomedical KGs. A notable trend across several papers [ 6 , 7 , 19 ] is the emerging role of LLMs in augmenting knowledge representation and reasoning. These works illustrate how LLMs can support ontology generation, improve diagnostic prediction, and enable information extraction when integrated with structured medical knowledge sources. Finally, the challenge of semantic interoperability and data harmonization is addressed in [ 8 , 20 , 21 ], including one of the top three papers.
